# Localized ST Elevations and PR Depressions in Systemic Lupus Erythematosus Pericarditis: An Unusual Case Presentation

**DOI:** 10.1155/carm/6633318

**Published:** 2026-02-17

**Authors:** Evan Derector, Madhurima S. Gundlapally, Nicholas R. Young

**Affiliations:** ^1^ Department of Internal Medicine, Cooper Medical School of Rowan University, Camden, New Jersey, USA, rowan.edu; ^2^ Department of Internal Medicine, Cooper University Healthcare, Camden, New Jersey, USA

## Abstract

Systemic lupus erythematosus (SLE) is a chronic autoimmune disease with frequent cardiac involvement. A 49‐year‐old male with a 20‐year history of SLE presented with a rash and lip swelling concerning for angioedema versus anaphylaxis. During bedside rounds, point‐of‐care‐ultrasound (POCUS) revealed a small posterior pericardial effusion, prompting a formal transthoracic echocardiogram (TTE). He subsequently developed acute substernal chest pain with ECG findings of isolated ST elevations and PR depressions, in the inferior leads (II, III, and aVF). Despite concerns for acute coronary syndrome (ACS), the team was reassured by POCUS, TTE, and negative troponins. He was diagnosed with SLE‐associated pericarditis and treated with NSAIDs, steroids, hydroxychloroquine, and methotrexate. This case highlights the importance of utilizing POCUS and physical examination skills to differentiate SLE‐associated pericarditis from ACS, particularly when ECG findings present in a localized, non‐diffuse pattern.

## 1. Introduction

Systemic lupus erythematosus (SLE) is an autoimmune multisystem disease involving overactivation of the innate and adaptive immune systems [1]. Aberrant B and T cells promote cytokine release, complement activation, and autoantibody production that affects multiple organs [1, 2]. While more common in women, SLE in men often presents more severely with systemic and cardiovascular involvement [1–3]. Cardiac manifestations, particularly pericarditis, are the most common and significantly contribute to morbidity and mortality [2, 4]. Over half of SLE patients present to care with cardiac symptoms, and autopsies reveal that up to 83% have pericardial inflammation although often asymptomatic [2]. When symptomatic, patients may present with chest pain, fatigue, a friction rub (a highly specific finding of pericarditis), and diffuse ST elevations and PR depressions on ECG, without localization to any specific coronary artery perfusion area [2, 4]. If untreated, SLE‐related pericardial disease can lead to serious complications like tamponade or constrictive pericarditis, requiring early diagnosis and treatment to reduce morbidity and mortality [2, 5].

## 2. Case Presentation

A 49‐year‐old male with a 20‐year diagnosed history of SLE, previously treated with prednisone and methotrexate, presented to our institution with a progressive facial rash and lip swelling. His presentation was concerning for angioedema versus anaphylaxis in the setting of multiple previous drug reactions to cephalosporins, sulfonamides, penicillin, and doxycycline. His SLE history included laboratory studies positive for antinuclear antibodies (ANAs), anti‐smooth muscle, and anti‐ribonucleoprotein antibodies, and clinical symptoms of a malar rash, pericarditis, pleuritis, and polyarthritis. In the months prior, he was treated with topical and oral antibiotics for diffuse rashes and skin infections and had multiple emergency department and outpatient visits for drainage of pustular lesions. His multidisciplinary care team discontinued immunosuppressants due to concern for worsening infections; however, the infections persisted, and he continued antibiotic treatment. He was prescribed another course of oral antibiotics for a new facial and anterior neck rash with superimposed infection but swelling worsened, prompting his presentation.

**FIGURE 1:**
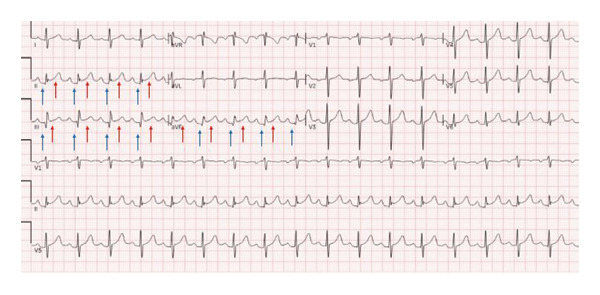
ECG findings: focal ST‐segment elevations with PR depressions in inferior leads (II, III, and aVF). Caption: 12‐lead ECG demonstrating localized acute inflammatory changes. Red arrows highlight focal, concave ST‐segment elevations, while blue arrows indicate concurrent PR‐segment depressions localized to the inferior leads (II, III, and aVF). Notably, there is an absence of reciprocal ST‐segment depressions in the high lateral leads (I and aVL), a key feature distinguishing this SLE‐associated pericarditis from an acute inferior myocardial infarction.

**FIGURE 2:**
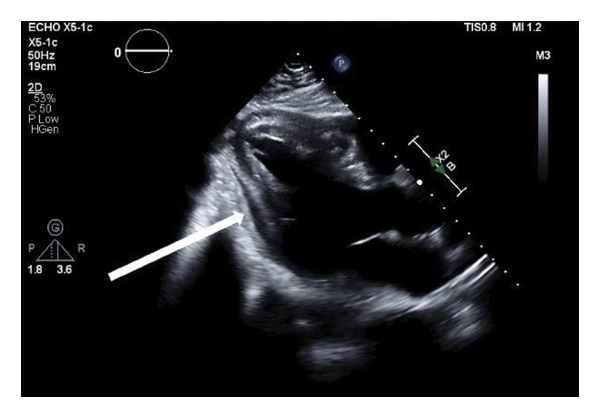
Echocardiogram: localized posterior pericardial effusion in parasternal long axis view. Caption: transthoracic echocardiogram (TTE) in the parasternal long‐axis (PSLAX) view. The white arrow identifies a localized, anechoic space posterior to the left ventricle, consistent with a trace‐to‐small pericardial effusion. This finding, identified initially via point‐of‐care ultrasound (POCUS) and confirmed by formal TTE, served as a primary diagnostic criterion for SLE‐associated pericarditis in the setting of elevated inflammatory markers.

His vitals (BP: 150/101 mmHg, pulse: 91 bpm, temperature: 98.2°F, and spO_2_: 98%) were stable, but his lip swelling and facial rash rapidly progressed. He was given Benadryl for a suspected allergic reaction, without signs of airway compromise. He was monitored overnight. During morning rounds, point‐of‐care‐ultrasound (POCUS) revealed a small posterior pericardial effusion, prompting a formal transthoracic echocardiogram (TTE). He subsequently developed acute substernal chest pain, diffuse arthralgias, hot flashes, and chills, prompting evaluation for symptom progression or a cardiac etiology. He became tachycardic (119 bpm) and hypotensive (107/66 mmHg); however, his airway remained patent, confirmed by CT neck, nasopharyngoscopy, and laryngoscopy. ECG showed isolated ST elevations and PR depressions in the inferior leads (II, III, and aV_*F*_), without reciprocal changes (Figure 1). While a pericardial friction rub was not present on exam, the diagnosis of acute pericarditis was confirmed based on the 2015 European Society of Cardiology (ESC) guidelines as the patient met three of the four required criteria: characteristic chest pain (sharp and pleuritic), ST‐segment elevation and PR‐segment depression on ECG, and a new pericardial effusion on imaging [6]. High‐sensitivity troponins were negative. A TTE confirmed normal ventricular function with a trace posterior pericardial effusion (Figure 2). He was treated with NSAIDs for pericarditis and given intramuscular methylprednisolone for the broader SLE exacerbation. He was started on prednisone and hydroxychloroquine for long term maintenance therapy. Laboratory results within 48 h prior to admission were positive for an ANA titer > 1:1280. Inflammatory makers were elevated, with a C‐reactive protein (CRP) of 5.48 mg/dL and an erythrocyte sedimentation rate (ESR) of 28 mm/hr. Complement levels were within normal limits prior to and during the admission. Additional studies were negative for any infectious or allergic etiology. His chest pain improved with treatment, and he was discharged with outpatient rheumatology follow‐up and a plan to continue hydroxychloroquine and add methotrexate for long‐term SLE management. At a 2‐week follow‐up visit with rheumatology, the patient reported complete resolution of chest pain, arthralgias, and constitutional symptoms. On physical examination, he was hemodynamically stable without a pericardial friction rub. While a repeat ECG was not obtained at this visit, the patient’s clinical improvement and lack of symptom recurrence while on maintenance hydroxychloroquine, methotrexate, folic acid, and a prednisone taper supported the resolution of acute SLE‐associated pericarditis.

## 3. Discussion

Although the literature estimates that over 50% of SLE patients present with cardiac symptoms and more than 30% experience pericarditis, a presentation involving ECG changes localized to a coronary artery perfusion region, yet indicative of a pericardial effusion and inflammation, has not been previously reported [2, 7]. Cardiac complications of SLE are critical to recognize as they are the leading cause of SLE‐related death [2]. SLE pericarditis often presents with chest pain, dyspnea, palpitations, and fatigue with a highly specific physical exam sign, a friction rub, and ECG findings of diffuse ST elevation and PR depression [8]. In contrast to horizontal ST elevations seen in acute myocardial infarction (MI), the ST changes in pericarditis are typically convex, with PR‐segment depression found in the same leads (Figure 1) [9–11]. These ECG findings result from repolarization abnormalities related to epicardial inflammation, usually diffuse, but in this case were localized to the inferior leads [9–11]. When present, ST elevations in pericarditis are usually more prominent in lead II than those in lead III, unlike acute STEMI [11]. This difference reflects injury orientation as in pericarditis, the epicardial inflammation creates an anterior‐leftward injury vector (aligned with lead II) whereas an inferior MI causes a rightward‐inferior vector due to transmural inflammation caused by coronary artery occlusion [11]. The localized findings in this case are likely due to regional, rather than diffuse, epicardial inflammation and the presence of an inferior pericardial effusion. Though this raised concern for acute coronary syndrome (ACS), negative troponins and the imaging results confirmed pericarditis, and early treatment was initiated to prevent complications.

The unique ECG findings in this patient highlight the importance of combining a clinical history, laboratory testing, POCUS, and cardiac imaging in evaluating atypical cardiac presentations. Prior literature has emphasized the need to recognize SLE cardiac involvement early to guide treatment [12–15]. SLE cardiac complications have been reported to mimic ventricular dysfunction, acute heart failure, constrictive pericarditis, infective endocarditis, valvular disease, ACS, and tamponade [12–15]. With the increased availability and use of POCUS, a highly sensitive and specific diagnostic tool for pericardial effusions, clinicians can supplement their diagnostic schema and physical exam with rapid bedside diagnostics [16]. In this case, the identification of a focal pericardial effusion on POCUS, with corresponding ECG changes and subsequent laboratory and imaging findings, helped confirm the diagnosis and prompted early treatment of SLE pericarditis.

Typically, these cases improve rapidly following the initiation of steroids, immunosuppressants, and supportive care; as seen here, but carry significant consequences if unrecognized [13, 16]. Since SLE masks itself as more commonly encountered cardiovascular conditions, these cases are frequently missed as clinicians fail to include SLE cardiac manifestations as a differential diagnosis (Table 1). However, missed SLE cardiac manifestations carry high rates of poor outcomes if treatment is not initiated in the early window period [12–15]. Therefore, clinicians should integrate detailed histories, focused physical exams, POCUS, serologic testing, and broad differentials to recognize and treat these cases early.

**TABLE 1:** Differential diagnosis for SLE pericarditis and typical clinical patterns [2, 17–29].

Feature	Lupus pericarditis	Inferior STEMI	Myopericarditis	Lupus myopericarditis	Coronary vasculitis in SLE
Clinical presentation	• Sharp, pleuritic chest pain • Worse when supine • Improved sitting forward (positional) • Pericardial friction rub	• Substernal chest pain • Nonpositional • Nonpleuritic • May radiate to the arm/jaw • Associated with dyspnea, diaphoresis	• Sharp, pleuritic chest pain, similar to pericarditis • May have preceding viral illness • Younger patients	• Unexplained tachycardia to fulminant heart failure • Dyspnea • Fatigue • Exercise intolerance • Often concurrent with pericarditis	• Typical or atypical anginal symptoms • Young women may present atypically • Men more typical presentation
ECG findings	• Widespread concave ST elevation with PR depression • No reciprocal changes	• Convex ST elevation in inferior leads (II, III, and aVF) • Reciprocal ST depression in anterior/lateral leads	• Diffuse ST elevation (may be less widespread than isolated pericarditis) • Non‐specific ST‐T changes	• Nonspecific findings, may show conduction abnormalities, arrhythmias	• ST elevation or depression in coronary distribution, may show Q waves
Troponin elevation	• Mild elevation in ∼30% of patients (subepicardial involvement) • Not prognostically significant	• Significantly elevated, progressive rise and fall	• Significantly elevated, indicates myocardial involvement but not negative prognostic marker	• May be normal or elevated, does not exclude the diagnosis	• Significantly elevated in acute MI pattern
Left ventricular function (LVEF)	• Preserved LVEF, normal wall motion	• RWMA in inferior territory, may have reduced LVEF	• May have global or regional dysfunction, usually preserved unless severe	• Reduced LVEF • Global or regional dysfunction	• RWMA in coronary distribution
Echocardiogram	• Pericardial effusion (60%) • Pericardial thickening • No myocardial dysfunction	• RWMA (inferior, inferolateral) • No effusion unless post‐MI	• Pericardial effusion may be present • Variable LVEF	• Reduced LVEF • Pericardial effusion common (69%) • May show subclinical dysfunction	• RWMA in vascular territory
Cardia MRI	• Pericardial edema and thickening • Myocardial LGE generally absent	• Myocardial edema in infarct territory • LGE in coronary distribution (subendocardial or transmural)	• Subepicardial basal and lateral myocardial edema • Patchy subepicardial or midventricular LGE	• Myocardial edema correlates with disease activity • Patchy noncoronary distribution of LGE	• Myocardial edema in affected territory • Subendocardial or transmural LGE in coronary distribution
Coronary angiography	• No obstructive CAD • Normal coronary arteries	• Acute plaque rupture or thrombotic occlusion in RCA or LCx	• Absence of obstructive CAD or acute plaque rupture	• Absence of obstructive CAD, may show diffuse vessel wall enhancement	• May show coronary arteritis, thrombosis, atherosclerotic disease, or normal with microvascular disease
Associated SLE features	• Early SLE manifestation • Associated with higher disease activity • Anti‐La antibodies	• Not specific to SLE • Traditional risk factors	• Not specific to SLE	• Occurs in 3%–15% of SLE patients, often during flares • Associated with active disease	• Accelerated atherosclerosis • Antiphospholipid antibodies • Young age • Active disease
Inflammatory markers	• Elevated CRP, ESR	• May be mildly elevated	• Mildly increased	• Elevated inflammatory markers with SLE activity	• Variable, may reflect SLE activity and atherosclerosis
BNP/NT‐proBNP	• Normal, unless tamponade	• Mildly to moderately elevated	• Mildly increased	• Elevated with heart failure	• Elevated with LV dysfunction

*Note:* Left circumflex artery (LCx).

Abbreviations: LGE, late gadolinium enhancement; RCA, right coronary artery; RWMA, regional wall motion abnormality; SLE, systemic lupus erythematosus.

This case highlights a rare presentation of SLE pericarditis with localized ECG changes mimicking an acute MI. Differentiating SLE pericarditis from other cardiac manifestations such as valvular disease or myocarditis is critical, as it can impact long‐term management. Given this patient’s history of SLE, positive autoantibodies, and acute symptoms, he was promptly treated with NSAIDs for pericarditis and restarted on immunosuppressive therapy for SLE, resulting in significant improvement. Early recognition and aggressive treatment of SLE cardiac manifestations are essential for favorable outcomes.

## Funding

This manuscript did not receive any funding from any source, including agencies in the public, commercial, or not‐for‐profit sectors.

## Disclosure

The remaining authors have nothing to disclose or relationships within the industry.

## Conflicts of Interest

The authors declare no conflicts of interest.

## Data Availability

The data that support the findings of this study are available on request from the corresponding author. The data are not publicly available due to privacy or ethical restrictions.
